# Atrial cardiopathy is associated with cerebral microbleeds in ischemic stroke patients

**DOI:** 10.3389/fneur.2022.982926

**Published:** 2022-09-01

**Authors:** David X. Zhao, Emma Gootee, Michelle C. Johansen

**Affiliations:** Department of Neurology, The Johns Hopkins University School of Medicine, Baltimore, MD, United States

**Keywords:** ischemic stroke, cerebral microbleeds, atrial cardiopathy, atrial dysfunction, magnetic resonance imaging

## Abstract

**Objective:**

Cerebral microbleeds (CMB) are small accumulations of hemosiderin associated with cerebrovascular risk factors, but whether they are associated with atrial cardiopathy is not known. The goal of this study is to determine, among ischemic stroke patients, the association between study-defined atrial cardiopathy and CMB presence, location, and number.

**Methods:**

Ischemic stroke patients admitted to Johns Hopkins (2015–2019) with transthoracic echocardiography and electrocardiography were included. Cerebral microbleeds were defined as small, round hypo-intensities on T2^*^ susceptibility weighted imaging or gradient recalled echo magnetic resonance imaging sequences. Atrial cardiopathy was defined as the presence of ≥1: left atrium diameter >4.0 cm (males) or >3.8 cm (females), PR interval >200 ms, or N-terminal pro-B-type natriuretic peptide >250 pg/ml. Binary/Ordinal logistic regression models were used to determine the association between atrial cardiopathy, and cerebral microbleed presence, location (lobar/deep), or number, each, adjusted for potential confounders.

**Results:**

Patients (*N* = 120) were mean age 60 years (range 22–98), 46% female, 62% black, and 39% were on anti-thrombotic medication at time of admission. 39 (32%) participants had ≥1 cerebral microbleeds. Forty-six (38%) patients had atrial cardiopathy. Atrial cardiopathy was associated with higher odds of having cerebral microbleeds (OR 2.50, 95% CI 1.02–6.15). Atrial cardiopathy was associated with lobar cerebral microbleeds (OR 2.33, 95% CI 1.01–5.37) in univariate analysis but not with deep cerebral microbleeds (OR 0.45, 95% CI 0.13–1.54), with neither association significant after adjustment. There was no difference in risk of having 1 vs. no cerebral microbleeds (RRR 2.51, 95% CI 0.75–8.37) and >1 cerebral microbleed vs none (RRR 2.57, 95% CI 0.87–7.60) among those with atrial cardiopathy.

**Conclusions:**

Atrial cardiopathy is associated with the presence, but not burden, of cerebral microbleeds in ischemic stroke patients. We cautiously suggest that atrial cardiopathy, either directly or through shared vascular risk, may contribute to the presence of CMB.

## Introduction

Cerebral microbleeds (CMB) are small hemorrhages that are described as small, round, or ovoid hemosiderin deposits on T2^*^-gradient recalled echo (GRE) or susceptibility weighted imaging (SWI) on cerebral magnetic resonance imaging (MRI) (1). CMB are now recognized to represent more than “asymptomatic lesions” found incidentally on brain MRI. At a minimum, they represent a state of cerebral small-vessel disease, and at the population level are known to co-occur with older age, stroke, intracranial hemorrhage, cognitive impairment, and all-cause mortality (1–6). Once CMB are detected, the impact that they have on the outcomes of patients with them are of great interest, as they may represent a potential biomarker for an underlying cerebrovascular disease state.

There has been increasing recognition of a state of atrial dysfunction that may be an important contributor to cerebral disease, apart from the existence of an atrial tachyarrhythmia, such as atrial fibrillation (AF) (7). Atrial cardiopathy is best defined as structural or functional changes in the left atrium that are characterized by either anatomical markers, or blood-based markers of atrial dysfunction (7, 8). Previously described markers of atrial dysfunction include left atrial enlargement and atrial strain on transthoracic echocardiogram (TTE) (8, 9). PR interval abnormalities on electrocardiogram (ECG) (10), and elevated N-terminal pro hormone brain natriuretic peptide (NT-proBNP) in the blood (11). Left atrial enlargement in particular has been associated with increased risk of AF and ischemic stroke (12).

Atrial cardiopathy, or atrial dysfunction, and its association with CMB has not been determined (13). In light of this, the primary aim of this study is to determine if atrial cardiopathy is associated with the presence, number and location of CMB in a cohort of ischemic stroke patients admitted to Johns Hopkins Hospital. A secondary aim is to determine if CMB are associated with stroke outcomes at 90 days.

## Materials and methods

### Study population

Participants for this study have been recruited as part of an ongoing prospective cohort study of ischemic stroke patients designed to describe atrial function and anatomy in patients with different stroke subtypes and cerebral imaging characteristics. This study has been approved by the Johns Hopkins Medicine Institutional Review Board and all patients provided informed consent. All procedures in this study were followed in accordance with the ethical standards of the Helsinki Declaration of 1975. The inclusion criteria for this study were adults admitted to Johns Hopkins Hospital (2015–2019) with ischemic stroke that had cerebral MRI, transthoracic echocardiogram, and electrocardiogram obtained at the time of admission. For this study, patients were excluded from analysis if there was a poor-quality MRI in which CMB could not be detected or did not have GRE or SWI MRI sequences. Additionally, patients were excluded if they had a history of prior intracranial hemorrhage had a history of dementia, or had a history of AF as defined below as these have all been previously associated with CMB.

Patient demographics, laboratory values, and other important vascular risk factors were collected at the time of stroke admission. These include smoking status (ever vs. never), history of hypertension, age, hemoglobin A1c, low density lipoprotein (LDL) levels, NIH stroke scale (NIHSS), antiplatelet medication use at time of admission and anticoagulant medication use at time of admission. AF was defined as either a documented prior history of AF, patient report during admission, a new diagnosis during the time of hospitalization or after discharge with cardiac monitoring for up to 6 months.

The modified Rankin Score (mRS) is scored from 0 (no residual symptoms) to 6 (death) to describe a patient's level of disability at 90 days after their ischemic stroke (14). For this study, certified raters called the patients by phone as is standard practice at the Johns Hopkins Comprehensive Stroke center. A mRS >2 was considered to be severe disability post-stroke for the purposes of this study, and therefore the dichotomization of the variable happened at this point.

### Cerebral microbleeds

**Figure 1:**
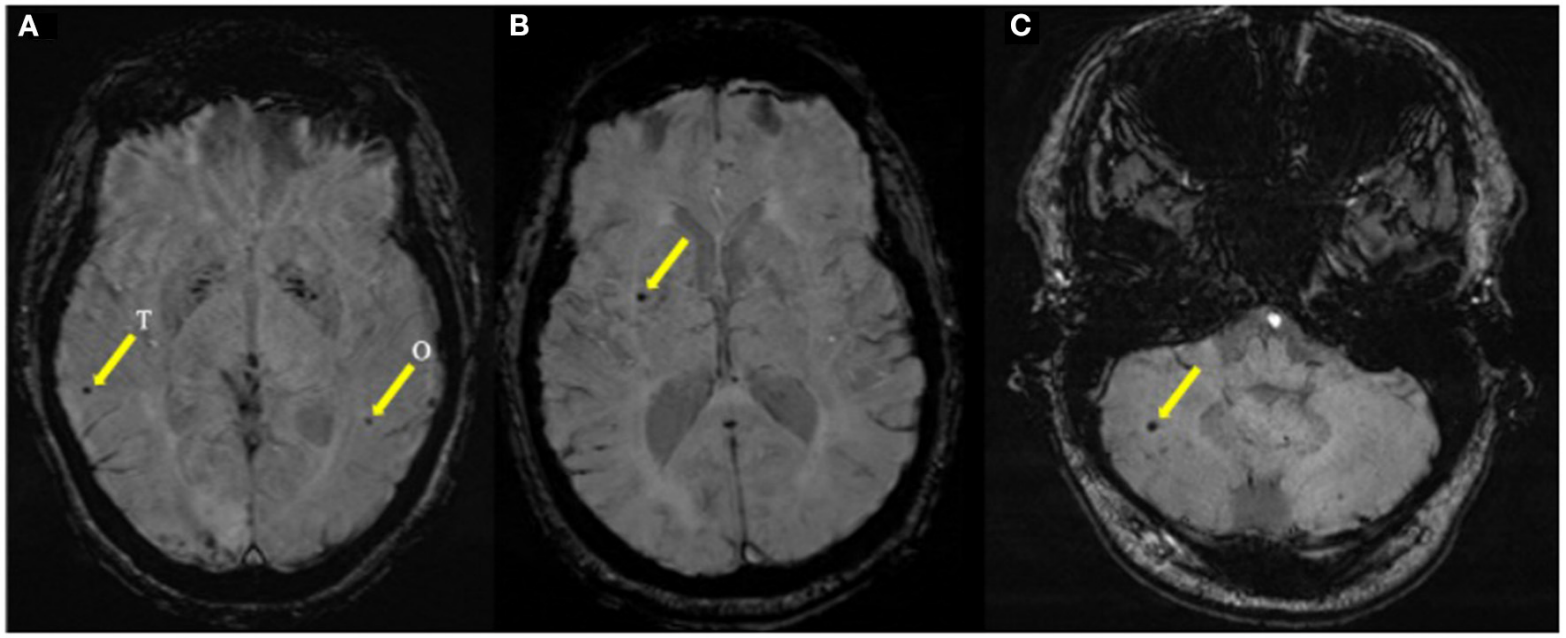
Representative MRI images of CMB in ischemic stroke patients. **(A)** Representative SWI sequence in a 72 years-old black female demonstrating two lobar CMB in left occipital lobe (O) and right temporal lobe (T). **(B)** Representative SWI sequence in a 66 years-old black male demonstrating a deep CMB in the basal ganglia. **(C)** Representative SWI sequence in a 56 years-old white male demonstrating an infratentorial CMB in the cerebellum.

CMB were defined per established criteria as round or ovoid hyperintensities <10 mm in diameter on T2^*^ SWI or GRE MRI sequences (1). CMB was determined using the protocol defined by Greenberg et al. (15) and determined by a trained imaging analyst and cerebrovascular neurologist masked to patient characteristics. Ten percent of the patients were evaluated independently by a second masked reviewer with an interrater reliability of 90%. In addition, Wardlaw's et al. (16) criteria were used to differentiate CMB from other mimics in the cerebral MRI such as lacune, small subcortical infarcts, calcium/mineralization artifact and perivascular space, and to exclude them. The Microbleed Anatomical Rating Scale (MARS) developed by Gregoire et al. (17) was used to count and categorize location of CMB (right or left; lobar, deep, or infratentorial). Examples of study MRI sequences are shown in Figure 1.

### Atrial cardiopathy

Atrial cardiopathy was defined as the presence of at least one of the following biomarkers of atrial dysfunction: enlarged left atrium diameter >3.8 cm/m^2^ in females or >4 cm/m^2^ in males on TTE (18), prolonged PR interval **≥**200 ms in ECG lead V_1_ (10), and elevated NT-proBNP serum levels >250 pg/ml (11).

### Statistical analysis

Multivariable logistic regression models were constructed to test the association between a state of atrial cardiopathy (independent variable) and the presence of CMB (dependent variable), or location of CMB when present (lobar, deep, infratentorial), each, in separate models. When considering the association between atrial cardiopathy and CMB burden, defined as having no CMB, 1 CMB, or >1 CMB, a multinomial logistic regression model was used. Interaction terms were included for age, and anti-thrombotic medication use, to determine if there was evidence of effect measure modification.

For our secondary analysis, we determined the association between the presence of CMB (independent variable) and post-stroke 90-day continuous mRS (dependent variable) by constructing a multivariable ordered logistic regression model, and a binary logistic regression model for binary mRS (0–2 vs. 3–6).

All regression models were adjusted in a stepwise fashion for potential confounders based on the literature (19–21). Specifically, model 1 adjusted for age, race, and sex. Model 2 adjusted for model 1 and current smoking, a history of hypertension, diabetes mellitus, LDL, and NIHSS. Model 3 adjusted for model 2 and any antithrombotic use (defined as anti-platelet or anti-coagulation use) at time of admission. All statistical analyses were performed using Stata v15.1 (22). Two-sided *p* < 0.05 was considered statistically significant.

## Results

### Patient characteristics

One hundred sixty-seven patients met the study inclusion criteria. Of these, 47 patients were excluded for the following reasons: eight had missing SWI/GRE MRI sequences, seven had poor quality SWI/GRE MRI sequences on which CMB could not be definitively determined, one did not have clinically diagnosed acute ischemic stroke, one had an MRI that demonstrated extensive intracranial hemorrhage as well as ischemic stroke, and 30 patients had prevalent AF. No patients had a prior history of dementia at the time of admission. Patient baseline characteristics are provided (Table 1). Thirty-nine percent of the patients were on anti-thrombotic (anti-coagulant or anti-platelet) medication at time of admission. The number of patients with at least one CMB was 39 (32%). Twenty-seven (23%) patients had ≥1 lobar CMB, 13 (11%) had ≥1 deep CMB, and nine (8%) had ≥ 1 infratentorial CMB.

**Table 1:** Patient characteristics.

	**Total (*n* = 120)**	**No atrial cardiopathy (*n* = 74)**	**Atrial cardiopathy (*n* = 46)**	***p*-Value**
Age, year, mean (SD)	59.61 (13.9)	56.3 (14.1)	65.0 (11.7)	**<0.001**
Female sex	55 (45.8)	30 (41)	25 (54)	0.14
Black race	74 (61.7)	44 (59)	30 (65)	0.53
Body mass index, kg/m^2^, mean (SD)	29.28 (7.1)	28.0 (6.5)	31.3 (7.6)	**0.013**
Ever smoker	67 (55.8)	38 (51)	29 (63)	0.21
Hemoglobin A1C	6.4 (1.9)	6.5 (2.2)	6.2 (1.2)	0.36
LDL, mg/dl	107.0 (42.3)	104.8 (39.0)	110.4 (47.2)	0.48
History of hypertension	95 (79.2)	55 (74)	40 (87)	0.098
Anti-platelet medication use	42 (35.0)	26 (35)	16 (35)	0.97
Anti-coagulation medication use	9 (7.5)	3 (4)	6 (13)	0.069
NIHSS ≥9	16 (13.8)	10 (14)	6 (13)	0.91
mRS ≥3^†^	16 (20.0)	7 (14)	9 (29)	0.11

Data are n (%) unless otherwise noted.

LDL, low-density lipoprotein; NIHSS, NIH Stroke Scale out of 42; mRS, modified Rankin Scale out of 6.

Definitions: Anti-Platelet Medication Use is defined as taking at least one of aspirin, Plavix, Effient, Brillinta, Aggrenox, or Ticlid at time of admission. Anti-Coagulation Medication Use is defined as taking at least one of heparin, warfarin, Xarelto, Pradaxa, Eliquis, or Lovenox at time of admission. Use is defined as taking at least one of Lipitor, Welchol, Zetia, Fenofibrate, Lopid, Lovastatin, Niacin, Crestor, Pravachol, or Zocor at time of admission.

Bold: Indicates statistical significance under the 2-sided p < 0.05 assumption.

### Atrial cardiopathy among patients with ischemic stroke

The mean LA diameter was 3.6 cm (SD 0.7), mean PR interval was 162.9 ms (SD 24.8), and mean NT-proBNP level was 833.7 pg/ml (SD 1264.6). Forty-six (38%) patients met criteria for atrial cardiopathy. Thirty-one (26%) patients had an enlarged LA diameter, 12 (10%) patients had an elongated PR interval, 18 (18%) patients had elevated serum NT-proBNP. Patients with atrial cardiopathy were older (65 vs. 56 years, *p* < 0.001) and had higher BMI [31.3 (SD 7.6) vs. 28.0 (SD 6.5), *p* = 0.013; Table 1].

### Associations between atrial cardiopathy and CMB presence and location

Among patients with atrial cardiopathy, the prevalence of CMB was 46%. The odds of having at least one CMB was 2.50 (95% CI 1.02–6.15, Model 3) times higher among patients with atrial cardiopathy compared to those without atrial cardiopathy (Table 2). There were no significant association between atrial cardiopathy and CMB location (lobar OR 2.68, 95% CI 0.98–7.32, Model 3; deep OR 0.46, 95% CI 0.11–2.01, Model 3). When investigating the possibility for effect modification of the association between atrial cardiopathy and CMB presence by age or anti-thrombotic medication use, we did not find the effect differed between older and younger patients (*p*-interaction = 0.10), or between those taking or not taking anti-thrombotic medication at the time of admission (*p*-interaction = 0.30).

**Table 2:** Multivariable logistic regression models demonstrating the association of atrial cardiopathy with CMB presence, and CMB location, among patients with ischemic stroke (*N* = 120).

**Outcome**	**Univariate**		**Model 1**		**Model 2**		**Model 3**	
	**OR**	**95% CI**	**OR**	**95% CI**	**OR**	**95% CI**	**OR**	**95% CI**
CMB Presence	**2.61**	**1.19**–**5.74**	2.05	0.90–4.70	**2.47**	**1.01**–**6.07**	**2.50**	**1.02**–**6.15**
Lobar CMB	**2.50**	**1.04**–**5.99**	2.15	0.85–5.45	2.69	0.99–7.33	2.68	0.98–7.32
Deep CMB	0.69	0.20–2.38	0.47	0.12–1.81	0.47	0.11–1.95	0.46	0.11–2.01

Adjustment models: Model 1 = age, race, sex. Model 2 = Model 1 + ever smoker, history of hypertension, diabetes mellitus, low-density lipoprotein (mg/dl), and National Institutes of Health stroke scale. Model 3 = Model 2 + anti-thrombotic medication use at time of admission (anti-coagulants and/or anti-platelet medications).

CI, confidence interval; CMB, cerebral microbleeds; OR, odds ratio, demonstrating the odds of any CMB vs. none.

Bold: Indicates statistical significance under the 2-sided p < 0.05 assumption.

### Associations between atrial cardiopathy and CMB number

There was no difference found in the risk of having exactly one CMB (RRR 3.20, 95% CI 1.09–9.37), or >1 CMB (RRR 2.24, 95% CI 0.37–5.85, Ref none), among those with atrial cardiopathy compared to those without atrial cardiopathy (Table 3).

**Table 3:** Multinomial logistic regression models demonstrating the association of atrial cardiopathy with CMB number among patients with ischemic stroke (*N* = 120).

**CMB Number (ref. none)**	**Univariate**		**Model 1**		**Model 2**		**Model 3**	
	**RRR**	**95% CI**	**RRR**	**95% CI**	**RRR**	**95% CI**	**RRR**	**95% CI**
One	**3.20**	**1.09**–**9.37**	2.26	0.72–7.06	2.57	0.78–8.47	2.51	0.75–8.37
Greater than one	2.24	0.37–5.85	1.95	0.71–5.32	2.51	0.85–7.41	2.57	0.87–7.60

Same adjustment models as Table 2. RRR, Relative Risk Ratio, demonstrating the relative risk of exactly one CMB vs. none, or the relative risk of greater than one CMB vs. none.

Bold: Indicates statistical significance under the 2-sided p < 0.05 assumption.

### Associations between CMB and post-stroke 90 day outcomes

Among 104 patients who had 90-day post-stroke mRS available for analysis, we did not find a significant association between CMB presence and mRS >2 (OR 1.01, 95% CI 0.31–3.37, Model 3) (Table S1). Considering that our prior outcome, atrial cardiopathy, may be on the causal pathway between having CMB and having a poor outcome after an acute ischemic stroke, we also excluded these participants in a sensitivity analysis, with no changes in our findings (results not shown).

## Discussion

In this study, we found among a cohort of ischemic stroke patients that a state of atrial cardiopathy is associated with a higher odds of having CMB on brain MRI, controlling for vascular risk factors. We believe that our findings are significant as CMB have been associated with an increased risk of poor functional outcomes, such as an increased risk of future intracranial hemorrhage (23) and poor cognitive function (24–26).

Atrial cardiopathy is frequently defined using biomarkers and cardiac imaging and is thought to represent a state of atrial dysfunction, which may suggest an increased risk of embolization, and thereby ischemic stroke (27). Although we readily acknowledge that our work did not directly study mechanisms, it is interesting to consider the cause behind our described association. It is likely that vascular risk factors play an important role in the development of atrial cardiopathy, which may lead to the development of CMB through mechanisms yet to be described or, these risk factors concurrently increase the risk of CMB. Hypertension, for example, has been demonstrated to be associated with both atrial disease, as well as the development of CMB (28, 29). Since atrial cardiopathy is likely caused by uncontrolled vascular risk factors, it may co-occur with CMB in patients who experience an ischemic stroke through shared vascular risk. Our work is unique in that it examined atrial cardiopathy and CMB in a group of patients with ischemic stroke, thereby representing an enriched cohort in which to study this association. While this study focused on CMB, there are other imaging markers of silent brain insult, or small vessel disease, that will likely also become increasingly important measures of brain health in the future among those with atrial cardiopathy (30–32). Aggressive control of vascular risk factors in these patients would likely not only be beneficial in secondary stroke prevention, but could potentially decrease the risk of further CMB, or other brain imaging markers, associated with a state of atrial cardiopathy, when present in ischemic stroke patients.

Following ischemic stroke, it is paramount to determine the cause of the stroke as this guides secondary stroke prevention; for example, stroke in the setting of AF necessitates anti-coagulation for secondary stroke prevention. However, anti-coagulation use is known to not only increase the risk of symptomatic intracranial hemorrhage (33), but some data suggests that the number of CMB in patients with AF increase when patients are placed on oral anti-coagulation (2, 34). However, subgroup analyses of the New Approach Rivaroxaban Inhibition of Factor Xa in a Global Trial vs. Aspirin to Prevent Embolism in Embolic Strokes of Undetermined Sources (NAVIGATE ESUS) trial found that while CMB are a marker of increased risk of recurrent stroke and mortality, they do not increase the risk of poor outcomes for patients on rivaroxaban vs. aspirin, which is encouraging (35).

Although there are similarities between a state of atrial cardiopathy and AF (36–38). there are currently no guidelines for anti-coagulation use for ischemic stroke patients who are found to have atrial cardiopathy (37, 38). The AtRial Cardiopathy and Antithrombotic Drugs In Prevention After Cryptogenic Stroke (ARCADIA) trial (8) and the Apixaban for Treatment of Embolic Stroke of Undetermined Source (ATTICUS) trial (39, 40) are specifically randomizing patients with biomarker driven atrial cardiopathy (though different criteria than our study) to anticoagulation vs. antiplatelet therapy and may help answer some of these questions. Atrial cardiopathy may increase subclinical risk of embolic stroke, and with the addition of the findings of this study that risk may be marked by CMB (41). Future studies should investigate the role of anticoagulation for atrial cardiopathy and also consider whether there are differences in the number of CMB.

The odds of having a more severe mRS >2, a sign of worse post-stroke outcomes, were not greater in ischemic stroke patients in our study who had at least one CMB compared to those without CMB. A study on non-cardiogenic minor ischemic stroke patients (NIHSS score <4) found that CMB count was independently associated with mRS>2, however, the participants in our study included those who had more severe ischemic stroke which may lead to poorer outcomes overall regardless of CMB burden (42). It may also be that mRS is primarily a measure of physical disability outcome, and therefore would not capture other potential long-term effects past 90 days, such as potential cognitive ramifications of CMB. mRS is routinely and accurately collected for ischemic stroke patients, but other measures of post-stroke disability have been found to be associated with CMB, such as tests of cognitive ability (43).

We readily acknowledge limitations to our study. This reflects data from a single center, although Johns Hopkins is a comprehensive stroke center with a wide referral network. We cannot determine causality from this analysis. Although we tried to account for the most important confounders in our analysis, we acknowledge the potential for residual confounding. While it is standard of care for patients on the stroke service to have a TTE or ECG during admission, only 25% of patients had a serum NT-proBNP measurement with the majority of patients having an elevated serum NT-proBNP thereby meeting the definition of atrial cardiopathy. There may have been bias by indication (tests only ordered under high clinical suspicion) so as a result, we excluded those patients who met criteria for atrial cardiopathy only based on an elevated NT-proBNP. In this sensitivity analysis, we still found an association between atrial cardiopathy and presence of CMB (OR 2.67, 95% CI 1.20–5.95, Model 3), suggesting that our analysis remains robust. Finally, P-wave terminal force, which is the exact measure used in ARCADIA, was not available in our patient population. We used PR interval since it has been shown to be strongly associated with cryptogenic stroke and is a reliable marker of LA function but we recognize that this may make our work less comparable to the work of others (10).

## Conclusion

In conclusion, we believe that our results suggest that a state of atrial cardiopathy is associated with an increased risk of CMB. While we are unable to determine causality or establish mechanisms with this work, ongoing efforts in this cohort, paired with the work of others, should continue to elucidate the importance of atrial cardiopathy on brain health.

## Data availability statement

The raw data supporting the conclusions of this article will be made available by the authors, without undue reservation.

## Ethics statement

The studies involving human participants were reviewed and approved by Johns Hopkins Medicine Institutional Review Board. The patients/participants provided their written informed consent to participate in this study.

## Author contributions

DZ and EG acquired the data. DZ also analyzed the data and drafted the manuscript. MJ analyzed the data, drafted and edited the manuscript and designed the study. All authors read and approved the submitted manuscript.

## Funding

DZ received funding for this work from the American Heart Association's Student Scholarship in Cerebrovascular Disease and Stroke. MJ received funding through the American Heart Association Career Development Award (#19CDA34660295) and the National Institute of Neurological Disorders and Stroke (#K23NS112459). The funding sources had no role in study design. The authors report no disclosures.

## Conflict of interest

The authors declare that the research was conducted in the absence of any commercial or financial relationships that could be construed as a potential conflict of interest.

## Publisher's note

All claims expressed in this article are solely those of the authors and do not necessarily represent those of their affiliated organizations, or those of the publisher, the editors and the reviewers. Any product that may be evaluated in this article, or claim that may be made by its manufacturer, is not guaranteed or endorsed by the publisher.
