# Supplementary material for: Atrial cardiopathy is associated with cerebral microbleeds in ischemic stroke patients
Source: Front Neurol. 2022 Sep 1;13:982926. doi: 10.3389/fneur.2022.982926 (PMC9475192; doi:10.3389/fneur.2022.982926)
Supplement: Supplementary file 1 [file Table_1.docx]

**Table S1.** **Ordered Logistic Regression Models Demonstrating the Association of CMB Presence and Modified Rankin Scale (N=80)**

|  | Univariate | | Model 1 | | Model 2 | | Model 3 | |
| --- | --- | --- | --- | --- | --- | --- | --- | --- |
|  | OR | 95% CI | OR | 95% CI | OR | 95% CI | OR | 95% CI |
| CMB Presence | 1.22 | 0.39-3.84 | 0.93 | 0.25-3.36 | 0.73 | 0.16-3.21 | 0.73 | 0.17-3.19 |

*Adjustment models: Model 1= age, race, sex. Model 2=Model 1 + ever smoker, history of hypertension, diabetes mellitus, low-density lipoprotein (mg/dL), and National Institutes of Health stroke scale. Model 3=Model 2 + anti-thrombotic medication use at time of admission (anti-coagulants and/or anti-platelet medications).

†Abbreviations and Explanations: mRS = Modified Ranking Scale, OR = Odds Ratio, demonstrating the odds of a severe mRS 3-6 versus mild mRS 0-2.
